# The Impact of Psychological Capital and Perceived Social Support on the Development of Problem Behaviors Among Rural Adolescents: A Cross-Lagged Study

**DOI:** 10.3390/bs16020264

**Published:** 2026-02-11

**Authors:** Zhiming Huo, Tingting Tan, Na Yang, Jie Wu

**Affiliations:** 1 Key Research Base of Humanities and Social Sciences of the Ministry of Education, Academy of Psychology and Behavior, Tianjin Normal University, Tianjin 300387, China; huozhiming@stu.tjnu.edu.cn (Z.H.); tantingting@stu.tjnu.edu.cn (T.T.); 2 Center of Cooperative Innovation for Assessment and Promotion of National Mental Health under Ministry of Education, Tianjin Normal University, Tianjin 300387, China; 3 Faculty of Psychology, Tianjin Normal University, Tianjin 300387, China; 4 Tianjin Labor Economics School (The Second Senior Technical School, Tianjin Human Resources and Social Security Bureau), Tianjin 300380, China; 5 Student Affairs Department of the Party Committee, Tianjin Normal University, Tianjin 300380, China; yangna@tjnu.edu.cn

**Keywords:** psychological capital, perceived social support, problem behaviors, rural adolescents, random-intercept cross-lagged panel model

## Abstract

Problem behaviors among rural adolescents remain a significant public health concern, yet the temporal roles of key psychosocial resources are not well understood. Grounded in Conservation of Resources theory and Problem Behavior Theory, this study examined the longitudinal associations between psychological capital, perceived social support, and problem behaviors among rural Chinese adolescents. A three-wave, one-year longitudinal design was conducted with 770 adolescents (49.86% male, Mage = 16.36, SD = 1.57). Random-intercept cross-lagged panel models were applied to disentangle stable between-person differences from within-person processes. At the between-person level, adolescents with higher overall psychological capital and perceived social support reported lower levels of problem behavior. At the within-person level, psychological capital showed a time-specific protective effect, with short-term increases predicting subsequent reductions in problem behavior, whereas problem behavior did not predict later psychological capital. In contrast, perceived social support demonstrated reciprocal associations with problem behavior: higher support predicted later decreases in problem behavior, while elevated problem behavior predicted subsequent declines in perceived support. These findings indicate that psychological capital and perceived social support operate through distinct temporal mechanisms and highlight the importance of early internal resource development and sustained relational support in rural adolescent populations.

## 1. Introduction

Adolescence is marked by profound physical, cognitive, and emotional transformations that render young people especially susceptible to behavioral difficulties. These problem behaviors fall into two broad categories: internalizing issues such as anxiety, depression, and social withdrawal, and externalizing difficulties including aggression, rule violations, and delinquent acts (Achenbach, 1966). More precisely, internalizing behaviors refer to problems that are primarily directed inward, manifesting as mood disturbances, excessive worry, somatic complaints, and social disengagement, whereas externalizing behaviors involve outwardly directed problems such as disruptive conduct, hostility toward others, and norm-violating actions (Achenbach et al., 2016). Contemporary research emphasizes that these two dimensions frequently co-occur and may share common underlying mechanisms related to emotion dysregulation and self-control deficits (Askari et al., 2022). Beyond their immediate impact on adolescent functioning, these behavioral patterns often persist into adulthood, affecting individual health, family stability, and broader social cohesion (Kulakow et al., 2024; Schön & Daseking, 2024). A recent nationwide survey in China found psychiatric disorders among 17.5% of children and adolescents, with significantly elevated rates in the 12–16 age bracket (Li et al., 2022), underscoring why preventing adolescent problem behaviors has become a pressing public health priority. What is still less settled is how protective resources work across time—whether the “protective effect” mainly reflects stable differences between adolescents, or whether short-term rises and drops within the same adolescent matter for later behavior. This distinction is not cosmetic; it changes what we treat as a realistic intervention target.

Within China’s youth population, rural adolescents face particularly acute challenges. Currently, 55.01 million adolescents reside in rural areas, representing over one-third of the nation’s youth (Lv et al., 2024). These young people are exposed to a confluence of risk factors: their communities typically have fewer economic resources, limited access to quality healthcare and mental health services, and educational systems stretched thin (Cui et al., 2024). Research consistently demonstrates that rural youth experience problem behaviors at substantially higher rates than their urban peers (B.-B. Chen et al., 2022; Y. Zhang et al., 2021), highlighting the necessity of identifying protective factors that can buffer risk. Yet intervention efforts remain fragmented, underscoring the need to clarify which protective factors are realistically modifiable and how they operate over time.

Conservation of Resources (COR) theory provides a robust framework for understanding this issue. COR theory posits that individuals strive to acquire, maintain, and protect valued resources—including psychological capital (PsyCap) and perceived social support (PSS)—to buffer against stress and adversity (Hobfoll, 1989). PsyCap, comprising hope, efficacy, resilience, and optimism, serves as an internal resource that enhances self-regulation and reduces maladaptive behaviors (Luthans et al., 2007). Research demonstrates that adolescents with higher PsyCap exhibit lower internalizing and externalizing problems by mobilizing positive coping strategies (Yıldırım et al., 2025). Similarly, PSS—defined as subjective appraisals of available support from family, peers, and teachers—functions as an external resource that satisfies relatedness needs and mitigates the impact of stressors on problem behaviors (Schüürmann & Goagoses, 2022; Zimet et al., 1988). In COR language, PsyCap can be treated as a relatively internalized “resource reservoir,” whereas PSS is closer to an “external channel” that depends on ongoing relationships—and that difference implies they may show different within-person dynamics across time.

The theoretical precision required to advance this field demands integration of COR theory with Problem Behavior Theory. COR theory proposes that protective resources operate through two temporal dynamics: “resource caravans,” whereby external support accumulates internal psychological capital over time, and “loss spirals,” wherein behavioral problems progressively deplete both internal and external resources (Hobfoll, 1989). Problem Behavior Theory (Jessor, 1991) further specifies that maladaptation emerges from imbalances between internal psychological systems and perceived environmental supports. Recent empirical applications of COR theory in adolescent research have substantiated these propositions. A cross-national study has shown that psychological capital, which is defined as an individual’s resource pool under the COR framework, can significantly predict the academic engagement of college students, and this effect size remains consistent in the samples from Romania and Serbia (Paloș et al., 2023). A longitudinal study conducted among Chinese college students found that psychological capital, as a key resource in the COR theory, has a negative predictive effect on anxiety through emotional regulation strategies (Z. Liu et al., 2024). This highlights the role of resource reserves in buffering psychological distress during the transition to adulthood. In the specific context of rural China, Ruan et al. (2024) examined over 36,000 left-behind children and adolescents and found that family-level resource deficits—including parental absence and inadequate caregiving arrangements—significantly increased the risk of depression. Their findings underscore that resource constraints operate more restrictively in rural settings where external support infrastructure is limited, highlighting the COR framework’s particular explanatory power for understanding rural youth vulnerability. Despite these theoretical propositions, empirical tests remain largely cross-sectional, leaving three critical gaps unaddressed: (1) whether these resources exhibit distinct temporal stability patterns, (2) whether their longitudinal links with problem behaviors reflect stable between-person differences or within-person change processes, and (3) whether PSS is involved in reciprocal “loss spiral” processes with problem behaviors in the way COR theory would anticipate.

### 1.1. Psychological Capital and Rural Adolescent Problematic Behaviors

Psychological capital (PsyCap) represents a higher-order positive psychological construct consisting of four core components: hope (goal-directed determination and pathways planning), self-efficacy (confidence in one’s ability to mobilize resources to succeed at challenging tasks), resilience (the capacity to recover from adversity), and optimism (positive attributional style about present and future success) (Luthans et al., 2007; Luthans & Youssef-Morgan, 2017). A recent comprehensive meta-analysis synthesizing findings from 474 independent samples demonstrated that PsyCap exhibits robust negative associations with psychological distress and maladaptive behaviors across diverse populations (Loghman et al., 2023). Among adolescent samples specifically, systematic review evidence indicates that higher PsyCap levels are consistently associated with reduced internalizing symptoms including depression and anxiety, as well as lower externalizing problems such as aggression and delinquency (Preston et al., 2023; Yıldırım et al., 2025).

Psychological capital influences problematic behaviors both directly and indirectly. Research indicates that psychological capital supports self-control capacity (Baumeister et al., 2007), with adolescents demonstrating stronger self-control when facing risk factors. Emotion regulation serves as a significant mediator, as optimism and hope components promote positive emotions and strengthen regulation capacities, reducing emotiona driven problematic behaviors (Qian et al., 2022). Recent longitudinal evidence from Chinese adolescent samples confirms that PsyCap demonstrates developmental plasticity during adolescence, with intervention studies showing that targeted PsyCap training can enhance hope, efficacy, resilience, and optimism while simultaneously reducing depressive symptoms and behavioral problems (Luo et al., 2025). Notably, a school-based HERO intervention study found significant improvements in psychological wellbeing among adolescent students, supporting the malleability of these psychological resources during this developmental period (Heikkila et al., 2024). From a theoretical standpoint, COR theory’s resource reservoir principle suggests a specific temporal pattern: once internalized through repeated positive experiences, psychological resources should exhibit substantial stability across moderate time intervals, as they represent consolidated cognitive-affective schemas rather than fluctuating emotional states (Hobfoll et al., 2018). Furthermore, adolescents with higher capital possess self-regulatory capacity to resist behavioral deviations (Luthans & Youssef-Morgan, 2017), so we would expect PsyCap to show a relatively “insulated” pattern over time—protecting later behavior more than being reshaped by short-term behavioral fluctuations.

### 1.2. Perceived Social Support and Rural Adolescent Problematic Behaviors

Perceived social support refers to an individual’s subjective evaluation of the availability and adequacy of support from their social network, encompassing emotional care, instrumental assistance, and informational guidance (Zimet et al., 1988). This construct is distinct from received support, as perceived support captures individuals’ cognitive appraisals of the support resources they believe would be available if needed, which research consistently demonstrates to be more predictive of psychological outcomes than objective measures of support received (Rueger et al., 2016). Among adolescents, perceived social support typically derives from three primary sources—family, peers, and teachers—each contributing uniquely to developmental outcomes (Y. Zhang et al., 2024). Contemporary theoretical frameworks, including the multi-source social support model, emphasize that these support sources may operate through different mechanisms and exhibit varying degrees of influence across developmental stages (Holt-Lunstad, 2022). As a critical protective factor, perceived social support plays a fundamental role in buffering adverse environmental influences and promoting adaptive functioning among adolescents. For rural adolescents confronting multiple ecological risks, understanding how perceived social support influences problematic behaviors becomes particularly important.

Teacher support represents a particularly salient protective resource within the school microsystem. Research demonstrates that supportive teacher-student relationships directly reduce adolescent problem behaviors while also fostering positive peer dynamics that further buffer behavioral difficulties (Guo et al., 2020). A recent meta-analysis examining teacher-delivered interventions found significant effects on reducing both internalizing symptoms and externalizing behaviors among school-aged youth, with effect sizes ranging from small to moderate (Aldabbagh et al., 2024). For Chinese rural adolescents specifically, studies have documented that perceived teacher emotional support operates through multiple pathways—directly reducing externalizing problems while simultaneously enhancing peer relationship quality and mental health outcomes (Zhu et al., 2024). Teachers occupy a unique position as adult figures who can compensate for parental absence common among left-behind children, providing academic guidance, emotional validation, and behavioral modeling that may be particularly consequential when family support is compromised.

Peer support assumes increasing developmental salience during adolescence as young people spend progressively more time with age-mates and rely on peer relationships to meet needs for belonging and identity validation. Longitudinal social network analyses reveal that peer influence on problem behaviors operates through multiple mechanisms including selection (adolescents choosing similar peers), socialization (peers becoming more alike over time), and behavioral contagion processes (Fortuin et al., 2015). A comprehensive meta-analysis synthesizing findings across 72 longitudinal studies confirmed medium-sized peer influence effects on both internalizing and externalizing problems during adolescence, with particularly strong socialization effects for externalizing behaviors (Giletta et al., 2021). Importantly, positive peer relationships characterized by mutual respect and prosocial norms serve protective functions—adolescents who experience high-quality friendships demonstrate fewer behavioral problems and greater resilience when facing adversity (Rubin et al., 2015). For rural youth facing limited extracurricular opportunities and geographic isolation, cultivating positive peer connections within school settings may represent an especially critical intervention target.

Empirical evidence consistently demonstrates negative associations between perceived support and adolescent problem behaviors (Schüürmann & Goagoses, 2022). However, the protective effects vary across support sources—family support deficiency exhibits the strongest predictive relationship with problem behaviors compared to school or peer support (Garnefski & Diekstra, 1997), underscoring the primacy of family microsystems in rural contexts. The stress-buffering hypothesis provides theoretical grounding for these effects, posits that social support attenuates the impact of stressful life events on negative outcomes by providing coping resources during high-stress conditions (Cohen & Wills, 1985). Recent meta-analytic evidence has substantiated this buffering mechanism, demonstrating that perceived social support shows stronger protective effects under conditions of elevated stress, particularly among adolescents facing multiple ecological risks (Rueger et al., 2016). A comprehensive review synthesizing findings across 116 studies further confirmed that perceived support from multiple sources—particularly family and peers—exerts significant direct effects on reduced internalizing and externalizing problems, with effect sizes ranging from medium to large (Chu et al., 2010). Moreover, negative support perceptions demonstrate stronger associations with emotional and behavioral problems than positive support. Critically, COR theory’s loss spiral corollary provides the theoretical rationale for bidirectional associations: when adolescents exhibit behavioral difficulties, they may trigger negative reactions from significant others—parental frustration, peer rejection, teacher disciplinary responses—thereby eroding the relational foundations upon which support perceptions rest (Hobfoll, 2001). Unlike psychological capital’s internalized nature, perceived social support depends on ongoing interpersonal transactions that behavioral problems can actively disrupt. So, for PSS, the “direction” is plausibly two-way in a way PsyCap is not.

However, the protective effects vary across support sources. Recent longitudinal research with Chinese adolescents has demonstrated that family support deficiency exhibits particularly strong associations with both internalizing and externalizing problems, while peer support becomes increasingly influential during the transition through adolescence (Xia et al., 2025; Y. Zhang et al., 2024). A cross-lagged panel study found bidirectional relationships between social support perceptions and prosocial behavior, suggesting that support from different sources may have cascading effects on behavioral development (Xia et al., 2025). Notably, research on Chinese left-behind children has demonstrated that positive teacher-student relationships and strong peer connections offer protective effects against mental health problems, with these school-based support sources partially compensating for reduced family support during parental migration (Feng et al., 2024). The interplay between support sources suggests that intervention strategies should adopt a multi-pronged approach, simultaneously strengthening family connections while building robust teacher support systems and fostering positive peer environments within rural school settings.

### 1.3. The Longitudinal Impact of Psychological Capital and Perceived Social Support on Problem Behaviors Among Rural Adolescents

Although psychological capital and perceived social support have been individually linked to reduced problem behaviors, existing research has predominantly relied on cross-sectional designs. This limitation is especially concerning for rural adolescents, given their elevated vulnerability compared to urban counterparts (Fellmeth et al., 2018; Tang et al., 2018). Research suggests psychological capital levels exhibit developmental fluctuations, particularly during the transition from early to middle adolescence when cognitive capacities and identity formation undergo rapid change (Luthans et al., 2006). Similarly, perceived social support demonstrates considerable instability during adolescence as peer relationships intensify and family dynamics shift.

The Process-Person-Context-Time (PPCT) model provides a comprehensive lens for examining how these protective factors operate across time (Bronfenbrenner & Ceci, 1994). Within this framework, psychological capital constitutes a “person” characteristic, while perceived social support reflects “process” dynamics within the microsystem. For rural Chinese adolescents, cultural emphases on collective harmony and family interdependence may shape how these factors function (J. Liu et al., 2020). Yet research integrating both protective mechanisms longitudinally remains surprisingly sparse. Recent advances in longitudinal methodology, particularly cross-lagged panel modeling and random-intercept cross-lagged approaches, now permit more rigorous examination of temporal precedence and reciprocal effects between protective factors and behavioral outcomes (Orth et al., 2021). These methodological innovations are especially valuable for disentangling within-person developmental processes from stable between-person differences, an important distinction when examining how psychological capital and perceived social support dynamically influence problem behavior trajectories. Because traditional CLPM conflates these two layers, “cross-lagged effects” can look stronger (or cleaner) than the real within-person process (Hamaker et al., 2015). What we need, especially in rural samples, is a model that explicitly separates trait-like differences from time-specific deviations.

Research on rural Chinese adolescents, particularly those experiencing parental migration and left-behind status, has consistently documented elevated mental health risks. A comprehensive meta-analysis of the mental health outcomes of rural left-behind children found that the incidence of depression, anxiety and behavioral problems was significantly higher compared with their non-left-behind peers (Zhao & Yu, 2016). Furthermore, a systematic review and meta-analysis published in The Lancet revealed that parental migration adversely affects multiple health domains in left-behind children, including mental health, substance use, and academic performance (Fellmeth et al., 2018). Recent large-scale research in rural China demonstrates that left-behind children face elevated mental health risks, with a study of 36,612 left-behind children finding significant associations between family structure, caregiving arrangements, and depressive symptoms (Ruan et al., 2024). Similarly, longitudinal research during the COVID-19 pandemic revealed that disadvantaged children in rural China experienced elevated rates of problem behaviors including non-suicidal self-injury and smartphone addiction, underscoring the urgent need to identify modifiable protective mechanisms in this population (Xie et al., 2023). These findings underscore the urgency of identifying protective mechanisms that can buffer against these risks in vulnerable rural adolescent populations.

Prior longitudinal investigations have three critical limitations. First, they predominantly examined single protective factors in isolation (Lai et al., 2021; Ma & Sheng, 2023), often leaving it unclear whether observed “effects” were driven by stable individual differences.

Second, existing work relied predominantly on unidirectional analytical frameworks (Shek & Liang, 2018), unable to detect bidirectional feedback loops that COR theory proposes. Third, longitudinal research has disproportionately focused on specific risk exposures rather than systematically examining how multiple protective resources jointly buffer against problem behaviors.

The present study advances beyond these limitations through three methodological innovations. First, we examine psychological capital and perceived social support simultaneously as complementary psychosocial resources, testing whether they function independently or synergistically. Second, we use three waves of data spanning one year to test temporal ordering and potential reciprocity. Third, we apply random-intercept cross-lagged panel models (RI-CLPMs) to disentangle stable between-person differences from within-person fluctuations, providing clearer evidence about change-like processes.

The present study addresses these limitations by applying random-intercept cross-lagged panel models (RI-CLPMs) across three waves, and by comparing two resource-behavior systems in parallel: PsyCap-problem behavior and PSS-problem behavior. This design lets us ask, with fewer hidden assumptions, whether PsyCap primarily operates as a temporally prior internal resource that is prospectively associated with later problem behavior and whether PSS is embedded in a reciprocal exchange that can be undermined by behavioral difficulties.

### 1.4. Current Study

This study focuses on middle adolescence (ages 14–19) for three developmentally grounded reasons. First, this period represents a critical phase when cognitive, affective, and behavioral regulatory systems undergo asynchronous maturation—a developmental disjunction creating heightened vulnerability to problem behaviors (Steinberg, 2005). Second, our selection of 6-month assessment intervals is calibrated to the tempo of adolescent resource fluctuation: intervals short enough to capture meaningful changes in context-dependent resources like social support, yet long enough to differentiate stable trait-like resources from transient emotional states (Crone & Dahl, 2012). Third, middle adolescence is when individual differences in resource accumulation become particularly consequential—behavioral problems consolidating during ages 14–16 predict substantially higher risks for school dropout, substance dependence, and criminal justice involvement (Moffitt, 1993).

Guided by COR theory and Problem Behavior Theory, and using RI-CLPMs to separate stable between-person differences from within-person fluctuations, we propose three hypotheses:

**H1.**  *Adolescents with higher overall psychological capital and higher overall perceived social support will exhibit lower overall levels of problem behavior across the one-year period*.

**H2.**  *When an adolescent reports higher-than-usual psychological capital at an earlier wave, they will report lower problem behavior at the subsequent wave; conversely, earlier problem behavior will not meaningfully predict later psychological capital*.

**H3.**  *Perceived social support will show reciprocal within-person links with problem behavior—higher-than-usual perceived support will predict subsequent reductions in problem behavior, while higher-than-usual problem behavior will predict subsequent decreases in perceived support (i.e., a loss-spiral process)*.

## 2. Materials and Methods

### 2.1. Participants and Procedure

This study employed a longitudinal design to dynamically examine problem behaviors among rural adolescents and their associated factors across three time points (T1, T2, and T3). Participants were seventh-, eighth-, tenth-, and eleventh-grade students at baseline (T1; June 2023) from five rural public secondary schools in Tianjin, China; across the one-year follow-up (T1–T3), these cohorts progressed to 8th, 9th, 11th, and 12th grades by T3 (June 2024). Following the National Bureau of Statistics’ compilation rules for the Statistical Zoning Code and the Urban-Rural Division Code (National Bureau of Statistics of China, 2009), we first matched each school to its Statistical Zoning Code and then used the corresponding Urban-Rural Division Code to confirm that all five schools were classified as rural community units (e.g., village/township codes such as 210/220), rather than urban subdistrict communities (e.g., 111/112). At the individual level, we recorded students’ hukou type and their current place of residence at the time of survey administration. A stratified random sampling method was used to ensure sample representativeness. Considering grade progression and cohort retention in this one-year longitudinal design, we recruited 7th-, 8th-, 10th-, and 11th-grade cohorts at T1 and limited follow-up to one year, because the 8th- and 11th-grade cohorts would enter graduation/examination years (9th and 12th grades) by T3, making longer follow-up difficult; accordingly, baseline 9th- and 12th-grade students were not sampled. The first wave of data collection (T1) was conducted in June 2023, with 900 questionnaires distributed and 840 valid responses collected. The second wave (T2) was conducted in December 2023, yielding 811 valid responses. The third wave (T3) was conducted in June 2024, resulting in 791 valid responses. A total of 770 rural adolescents completed all three waves of the survey, including 374 males (49.86%), with ages ranging from 14 to 19 years (M = 16.36, SD = 1.57). The distribution of participants by gender and grade at T3 (June 2024) is presented in Table 1.

### 2.2. Measures

#### 2.2.1. Strengths and Difficulties Questionnaire (SDQ)—Student Version

The Strengths and Difficulties Questionnaire (SDQ) for students (R. Goodman, 1997) was employed to evaluate problem behaviors among rural adolescents. Following established research practices (Gui et al., 2021; Stoddard-Bennett et al., 2023), we selected three subscales—emotional symptoms, hyperactivity/inattention, and conduct problems—to assess adolescent problem behaviors. Representative items include: “I worry a lot” (emotional symptoms); “I am restless, I cannot stay still for long” (hyperactivity/inattention); and “I get very angry and often lose my temper” (conduct problems). Each item is rated on a three-point Likert scale, with higher scores reflecting greater problem behavior severity. The internal consistency coefficients across three surveys were 0.76, 0.70, and 0.8.

Our decision to use three subscales rather than the standard four-subscale total difficulties score was based on several considerations. First, the peer problems subscale has consistently demonstrated the weakest psychometric properties across international studies. Du et al. reported that the reliability of peer problems in Chinese adolescents was particularly concerning (Du et al., 2008). A systematic review indicated that Cronbach’s alpha for the peer problems subscale in Chinese samples ranged from 0.30 to 0.70, substantially lower than that of the other SDQ subscales (C.-b. Chen & Chen, 2017). Likewise, a cross-cultural study found that the alpha for peer problems in five European countries ranged from 0.27 to 0.52, the lowest among all SDQ scales (Essau et al., 2012). Second, our approach aligns with Stoddard-Bennett et al. (2023), who selectively used emotional symptoms, hyperactivity, and conduct subscales in their cross-national study. The SDQ developer also acknowledges that alternative subscale configurations may be appropriate depending on research purposes (A. Goodman et al., 2010). Third, the peer problems subscale may overlap conceptually with our perceived social support measure, potentially introducing multicollinearity. Our composite score reliability (0.70–0.81) meets conventional thresholds for research instruments (Tavakol & Dennick, 2011), and the SDQ has demonstrated good convergent validity with the Youth Self-Report in Chinese populations (Yao et al., 2009).

#### 2.2.2. Positive Psychological Capital Questionnaire (PPCQ)

We used the 26-item Positive Psychological Capital (PPCQ) scale (K. Zhang et al., 2010). This scale comprises four dimensions: self-efficacy, resilience, optimism, and hope. Representative items for each dimension include: “I feel confident analyzing a long-term problem to find a solution” and “I feel confident presenting information to a group of people” (self-efficacy, 7 items); “I can get through difficult times because I have experienced difficulty before” and “I usually take stressful things in stride” (resilience, 7 items); “When things are uncertain, I usually expect the best” and “I always look on the bright side of things” (optimism, 6 items); and “I can think of many ways to reach my current goals” and “Right now I see myself as being pretty successful” (hope, 6 items). Participants rated each item on a 7-point Likert scale, ranging from “Completely Disagree” to “Completely Agree.” An average score across all items was computed to indicate the overall psychological capital level, with higher scores reflecting greater psychological capital. In the three surveys, the internal consistency coefficients were 0.91, 0.89 and 0.9. These reliability coefficients exceed the 0.85 threshold typically reported in Chinese adolescent samples (K. Zhang et al., 2010), indicating excellent internal consistency.

#### 2.2.3. Perceived Social Support Scale (PSSS)

We assessed perceived social support using the Perceived Social Support Scale (Zimet et al., 1988). This scale has been validated among Chinese adolescent populations and demonstrated robust psychometric properties (Gu et al., 2024). The PSSS comprises 12 items across three dimensions: family support, friend support, and support from others. Representative items for each subscale include: “My family really tries to help me” and “I can talk about my problems with my family” (family support, 4 items); “I can count on my friends when things go wrong” and “I have friends with whom I can share my joys and sorrows” (friend support, 4 items); and “There is a special person who is around when I am in need” and “There is a special person in my life who cares about my feelings” (significant other support, 4 items). Participants rate each item on a 7-point Likert scale ranging from “Strongly Disagree” to “Strongly Agree,” with higher mean scores indicating greater perceived social support. In the three surveys, the internal consistency coefficients were 0.92, 0.98 and 0.9. These coefficients are consistent with the excellent reliability typically observed for the PSSS across diverse populations (Zimet et al., 1988).

### 2.3. Collection and Analysis of Data

The study received ethical clearance from the Ethics Committee of the Academy of Psychology and Behavior at Tianjin Normal University. We obtained permissions from school principals, class instructors, and legal guardians before initiating data collection. All adolescents participated on a voluntary basis and provided written informed consent after being briefed on study objectives and procedures. Graduate-level psychology students with specialized training supervised the survey administration in classroom settings, clarifying instructions and distributing materials. Participants were informed they could withdraw at any point without consequences.

Graduate assistants handled data entry with systematic verification protocols to maintain data integrity. We performed descriptive analyses and examined bivariate correlations through SPSS 27.0. To detect potential common method variance, we applied Harman’s single-factor approach (Podsakoff et al., 2003). Longitudinal structural models were estimated in Mplus 8.11 using robust maximum likelihood (MLR) to accommodate potential non-normality (Muthén & Muthén, 2017). Rather than relying on a traditional CLPM, we fitted random-intercept cross-lagged panel models (RI-CLPMs) to separate stable between-person differences (random intercepts) from within-person fluctuations (time-specific deviations) in the focal constructs (Hamaker et al., 2015; Orth et al., 2021). Consistent with our conceptual distinction between internal resources (psychological capital) and external relational resources (perceived social support), RI-CLPMs were estimated separately for the psychological capital-problem behavior (PC-PB) system and the perceived social support-problem behavior (PSS-PB) system.

Multiple goodness-of-fit metrics evaluated model adequacy: chi-square/degrees of freedom ratio, comparative fit index (CFI), root mean square error of approximation (RMSEA), and standardized root mean square residual (SRMR). We adopted widely recognized benchmarks: CFI values at or above 0.95, RMSEA at or below 0.08, and SRMR at or below 0.08 signify adequate model-data correspondence (Hu & Bentler, 1999; Tabri & Elliott, 2012). At the within-person level, autoregressive paths indexed the carryover of deviations from an adolescent’s own expected level over time, whereas cross-lagged paths tested whether higher-than-usual resources (or problem behavior) predicted subsequent deviations in the other construct across the following six months. At the between-person level, covariances between random intercepts captured stable associations across the one-year period. In this specification, the cross-lagged coefficients are interpreted as prospective within-person effects (i.e., change-like dynamics), rather than being dominated by between-person differences that can inflate or obscure temporal interpretation in traditional CLPMs (Hamaker et al., 2015; Orth et al., 2021).

## 3. Results

### 3.1. Common Method Bias Analysis

To examine common method bias, this study conducted Harman’s single-factor test. The results were as follows: (1) At Time 1, 11 factors had initial eigenvalues greater than 1, with the first factor accounting for 31.19% of the variance, below the 40% threshold; (2) At Time 2, 10 factors had initial eigenvalues greater than 1, with the first factor accounting for 35.01% of the variance, also below the 40% threshold; (3) At Time 3, 8 factors had initial eigenvalues greater than 1, with the first factor accounting for 41.13% of the variance, slightly above the 40% threshold but well below 50%. These results indicate that common method bias was not a serious concern in this study (Lindell & Whitney, 2001).

### 3.2. Descriptive Statistics and Correlations

Table 2 presents the correlations among problem behaviors (including internalizing, externalizing, and total scores), psychological capital, and perceived social support across three time points (Time 1, Time 2, and Time 3). Overall, the variables demonstrated systematic correlation patterns both within and across waves, with most relationships being statistically significant (*p* < 0.01).

Within each wave, problem behavior was significantly negatively correlated with both psychological capital and perceived social support. This pattern remained largely consistent across the second and third assessments, indicating stable negative concurrent associations between these psychosocial resources and problem behavior.

Cross-time analysis revealed moderate to strong positive correlations for problem behaviors across different assessments (e.g., first time internalizing with second time internalizing: *r* = 0.54; first time total problem behavior with third time total problem behavior: *r* = 0.32), suggesting a degree of stability in problem behaviors over time. Psychological capital and perceived social support also showed significant positive correlations across time points (e.g., second time psychological capital with third time psychological capital: *r* = 0.66; second time social support with third time social support: *r* = 0.66), indicating continuity in these positive psychological resources across stages.

Furthermore, significant negative correlations were observed between problem behaviors and both psychological capital and social support across different time points. For instance, first time problem behaviors correlated negatively with second time psychological capital (*r* = −0.44 to −0.41) and third time psychological capital (*r* = −0.40 to −0.33). Similarly, perceived social support was negatively correlated with problem behaviors at various stages (e.g., first time social support with third time problem behaviors: *r* = −0.21 to −0.29).

It is noteworthy that some correlations involving externalizing problem behavior at the second time point (Variable 2.2) were weak or non-significant (e.g., with first time problem behavior dimensions: *r* = 0.05–0.06), suggesting that externalizing problem behavior may have weaker associations with other variables in certain contexts.

In summary, problem behaviors, psychological capital, and perceived social support showed systematic and significant correlations both within and across the three time points, supporting their interrelatedness and dynamic associations in this longitudinal context.

### 3.3. Cross-Lagged Analysis of Problem Behavior, Psychological Capital, and Perceived Social Support

To examine the within-person reciprocal associations between psychosocial resources and problem behavior while accounting for stable between-person differences, random-intercept cross-lagged panel models (RI-CLPMs) were estimated separately for psychological capital–problem behavior (PC–PB) and perceived social support–problem behavior (PSS–PB). All models were estimated using maximum likelihood estimation with robust standard errors (MLR).

**Figure 1:**
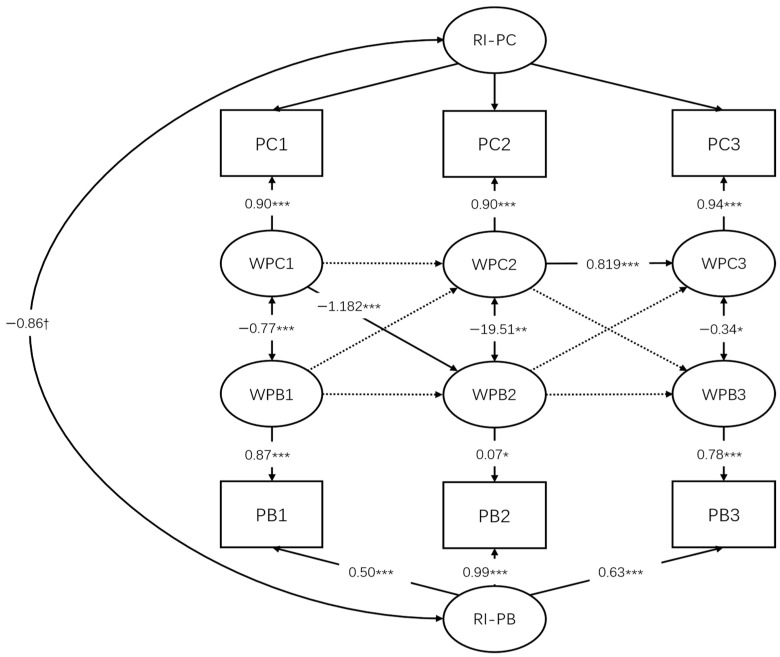
Random Intercept Cross-Lagged Panel Model of Psychological Capital and Problem Behavior. * *p* < 0.05,** *p* < 0.01, *** *p* < 0.001, 0.05 < *p* < 0.1,^†^.

For the PC–PB RI-CLPM (Figure 1), model fit evaluation focused primarily on the standardized root mean square residual (SRMR), as the chi-square test of model fit was not considered informative due to the presence of a scaling correction factor. The SRMR value was 0.087, indicating a marginally acceptable fit for a complex longitudinal structural model. Information criteria further supported the adequacy of the specified model.

Before examining within-person dynamics, between-person associations were inspected. At the between-person level, substantial individual differences were observed in the random intercept for problem behavior (Var = 8.05, SE = 0.59, *p* < 0.001), whereas the variance of the random intercept for psychological capital was not statistically significant (Var = 126.37, SE = 158.74, *p* = 0.426). The random intercepts of psychological capital and problem behavior were negatively associated (cov = −27.52, SE = 4.33, *p* < 0.001; *r* = −0.86), indicating that adolescents with higher overall psychological capital tended to exhibit lower overall levels of problem behavior across time.

At the within-person level, psychological capital exhibited partial temporal stability. A significant autoregressive effect was observed from Wave 2 to Wave 3 (*β* = 0.82, SE = 0.25, *p* = 0.001), whereas the autoregressive effect from Wave 1 to Wave 2 was not statistically significant after controlling for stable between-person differences (*β* = 0.48, SE = 0.34, *p* = 0.161). For problem behavior, autoregressive effects were not statistically significant across adjacent waves, suggesting limited within-person stability once trait-like individual differences were accounted for.

With respect to cross-lagged effects, although the estimated coefficients from psychological capital to subsequent problem behavior differed numerically across intervals, formal equality constraints imposed on the cross-lagged and autoregressive parameters were not rejected. Wald tests indicated no systematic differences in these parameters across the Wave 1→Wave 2 and Wave 2→Wave 3 intervals. Accordingly, the cross-lagged paths from psychological capital to problem behavior were not interpreted as evidence of time-varying or stage-dependent effects. Overall, the results provided limited evidence for a within-person cross-lagged predictive effect of psychological capital on subsequent problem behavior in this model.

In the opposite direction, within-person deviations in problem behavior did not significantly predict later psychological capital from Wave 1 to Wave 2 (*β* = −0.001, SE = 0.24, *p* = 0.998) or from Wave 2 to Wave 3 (*β* = 0.07, SE = 0.07, *p* = 0.373).

Concurrent within-wave associations were consistently negative, such that higher-than-usual psychological capital was associated with lower-than-usual problem behavior at Wave 1 (*r* = −0.77, SE = 0.09, *p* < 0.001), Wave 2 (*r* = −0.37, SE = 0.04, *p* = 0.003), and Wave 3 (*r* = −0.34, SE = 0.17, *p* = 0.043).

A model estimation warning indicated that the latent covariance matrix was not positive definite, involving the within-person problem behavior factor at Wave 2; accordingly, its residual variance was constrained to a small positive value to aid numerical stability. The substantive interpretation focuses on the pattern of within-person autoregressive and cross-lagged paths reported above.

**Figure 2:**
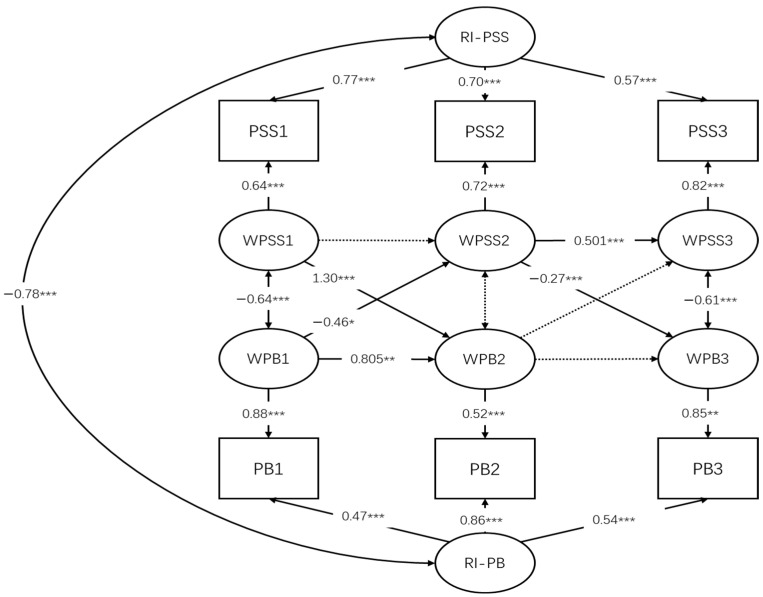
Random Intercept Cross-Lagged Panel Model of Perceived Social Support and Problem Behavior. * *p* < 0.05,** *p* < 0.01, *** *p* < 0.001.

For the PSS–PB RI-CLPM (Figure 2), multiple fit indices were available. The model demonstrated excellent comparative fit, with a Comparative Fit Index (CFI) of 0.982 and a low SRMR value of 0.050. Although the chi-square test was statistically significant and the RMSEA value exceeded conventional cutoff criteria (RMSEA = 0.122), these indices are known to be sensitive to small degrees of freedom and large sample sizes. In models with very low degrees of freedom, RMSEA may overestimate model misfit even when other indices indicate good fit. Taken together, the overall pattern of fit indices suggested that the RI-CLPM provided an adequate representation of the data.

At the between-person level, perceived social support and problem behavior were negatively associated, indicating that adolescents who generally perceived higher levels of social support tended to exhibit lower overall levels of problem behavior across the study period. Substantial individual differences were observed in the random intercepts of both constructs, supporting the presence of stable between-person variability.

At the within-person level, perceived social support demonstrated moderate temporal stability across adjacent waves. Cross-lagged analyses indicated that within-person changes in perceived social support were prospectively associated with subsequent changes in problem behavior, and the direction of this association differed across intervals. Formal equality testing further indicated that these cross-lagged effects differed significantly across intervals (Wald χ^2^(1) = 24.948, *p* < 0.001), with a positive effect observed from Wave 1 to Wave 2 (*b* = 0.238, *p* < 0.001) and a negative effect from Wave 2 to Wave 3 (*b* = −0.107, *p* < 0.001).

In the opposite direction, within-person elevations in problem behavior predicted subsequent decreases in perceived social support across waves, indicating a reciprocal within-person process. Concurrent within-wave associations between perceived social support and problem behavior were consistently negative, such that higher-than-usual perceived social support was associated with lower-than-usual problem behavior at each measurement occasion.

Across the two RI-CLPMs (PC–PB and PSS–PB), a common pattern emerged in that both psychosocial resources (psychological capital and perceived social support) were negatively related to problem behavior at the between-person level and showed negative within-wave associations, indicating that individuals who generally (and momentarily) reported higher resources tended to exhibit lower problem behavior. Notably, the PSS–PB model showed more consistent evidence for within-person cross-lagged associations over time, whereas the PC–PB model showed limited and less consistent evidence for within-person cross-lagged effects once stable between-person differences were accounted for.

## 4. Discussion

This year-long investigation examined the dynamic associations between psychological capital, perceived social support, and problem behaviors among rural Chinese adolescents using random-intercept cross-lagged panel models (RI-CLPMs). By separating stable between-person differences from within-person fluctuations over time, the present study provides a more refined understanding of how internal psychological resources and external social resources relate to adolescents’ behavioral adjustment. The findings reveal that psychological capital and perceived social support operate through distinct temporal mechanisms, highlighting the importance of distinguishing trait-like individual differences from dynamic intra-individual processes in rural youth development.

At the between-person level, both psychological capital and perceived social support were negatively associated with problem behavior, indicating that adolescents who generally reported higher psychosocial resources tended to exhibit lower levels of behavioral difficulties across the study period. This pattern aligns with prior research documenting the protective role of psychological capital and social support in adolescent adjustment (Finch et al., 2020; Rodríguez-Meirinhos et al., 2020; Xia et al., 2025). Importantly, these between-person associations reflect relatively stable individual differences rather than dynamic processes unfolding within individuals over time.

At the within-person level, psychological capital demonstrated a time-specific protective effect on problem behavior. Specifically, temporary increases in psychological capital at the initial assessment predicted subsequent reductions in problem behavior six months later, whereas this effect did not persist across later waves. This finding refines previous cross-lagged evidence by suggesting that the protective role of psychological capital may be most salient during early stages of adolescence, when self-regulatory capacities and coping resources are still consolidating. Consistent with conservation of resources theory (Hobfoll, 1989), adolescents who experienced short-term gains in hope, efficacy, resilience, and optimism may have been better equipped to manage developmental stressors and regulate behavioral impulses during this sensitive period. The attenuation of this effect over time may indicate that once behavioral patterns stabilize, additional increases in psychological capital exert diminishing marginal influence on subsequent behavioral change. Nevertheless, these within-person cross-lagged associations in an observational design should be interpreted as prospective (time-ordered) links rather than causal effects, and they may be influenced by unmeasured time-varying confounding.

In contrast, perceived social support exhibited more consistent and dynamic within-person associations with problem behavior. Increases in perceived social support predicted subsequent reductions in problem behavior across multiple waves, supporting stress-buffering frameworks that emphasize the role of interpersonal resources in mitigating behavioral risk (Cohen & Wills, 1985). Moreover, problem behavior also predicted subsequent decreases in perceived social support, revealing a reciprocal within-person process whereby behavioral difficulties erode adolescents’ access to supportive relationships. This bidirectional pattern is consistent with resource loss spirals articulated in conservation of resources theory (Hobfoll, 2001) and extends prior longitudinal findings documenting reciprocal associations between social support and adolescent adjustment (Xia et al., 2025; Y. Zhang et al., 2024). Relatedly, because perceived social support and problem behavior were assessed via self-report, reciprocal associations may partly reflect shared method variance or perception-based reporting; future studies using multi-informant or behavioral indicators are needed.

The contrast between psychological capital and perceived social support highlights their complementary yet distinct roles in rural adolescent development. Psychological capital appears to function primarily as an internal, time-sensitive buffer that shapes early behavioral trajectories, whereas perceived social support operates as a relational resource that both protects against and is vulnerable to behavioral difficulties. This distinction may be particularly salient in rural contexts, where structural constraints such as parental migration, limited mental health services, and reduced educational resources restrict adolescents’ access to alternative support systems (She et al., 2022; Wang et al., 2019). These constraints may be reflected in adolescents’ perceived social support (as measured here) by narrowing the availability of reliable family, teacher, and peer resources, and may amplify the consequences of support erosion following behavioral problems. Because these cultural and structural conditions were not directly measured in the present study, we treat them as contextual interpretation rather than tested mechanisms.

These findings may have practical implications for prevention and intervention efforts in rural settings. Programs aimed at enhancing psychological capital may be particularly beneficial when implemented early in adolescence, before maladaptive behavioral patterns become entrenched. To improve feasibility in resource-limited rural schools, such PsyCap-enhancement activities could be delivered through brief, manualized classroom modules (e.g., goal-setting, problem-solving, strengths reflection) embedded in routine class meetings or moral education courses and facilitated by trained homeroom teachers or school counselors. At the same time, interventions targeting problem behaviors may prioritize the preservation and repair of adolescents’ social support networks, given the observed reciprocal erosion processes. Feasible approaches include regular teacher–student mentoring check-ins, structured peer-support activities, and home–school communication plans (including phone/online contact with migrant parents or caregivers) to prevent support erosion following behavioral problems. School- and community-based initiatives that integrate psychological capital training with family–school–community collaboration may offer promising avenues for promoting adaptive development among rural youth (Finch et al., 2025; Li et al., 2022). These implementation suggestions are offered as practical extensions of our findings and should be tested in future intervention research. However, these practical implications should be interpreted cautiously given the observational design and the sample drawn from a single rural county; replication and intervention trials in diverse rural regions are needed.

Several limitations warrant consideration. Although the RI-CLPM approach improves temporal interpretation by separating within-person and between-person effects, it does not establish causality; unmeasured time-varying confounding and alternative explanations may remain. We also did not directly measure rural structural conditions (e.g., parental migration status, family socioeconomic resources, school service availability) or cultural orientations; therefore, our discussion of these factors is intended as contextual interpretation rather than an empirical test of their effects on psychological capital, perceived social support, or problem behavior. The reliance on self-report measures may also introduce shared method variance. Although the internal consistency of the SDQ-based composite was acceptable in our sample (Cronbach’s alpha = 0.70–0.81 across waves), measurement error cannot be fully ruled out and may have attenuated the estimated associations; future studies should replicate these findings using multi-informant assessments and latent-variable models that explicitly account for measurement unreliability. Additionally, the sample was drawn from a single rural county, limiting generalizability across diverse rural contexts in China. Future research should employ multi-informant designs, extend follow-up periods, and examine whether the observed within-person processes generalize across different cultural and socioeconomic settings. Despite these limitations, the present study advances understanding of how internal psychological resources and external social resources dynamically interact to shape problem behavior among rural adolescents.

More specifically, while RI-CLPMs help control for stable (time-invariant) between-person differences, the estimated within-person cross-lagged paths may still be biased by omitted time-varying factors that co-occur with changes in psychological capital, perceived social support, and problem behaviors. For example, developmental maturation, school transitions and academic stressors, family events (e.g., changes in caregiving arrangements or parental presence), and other contextual shocks may simultaneously influence adolescents’ resources and behavioral adjustment across the 6-month intervals, creating spurious prospective associations or obscuring true effects. Accordingly, the temporal ordering observed here should be interpreted as longitudinal associations rather than definitive causal effects. Future studies could strengthen causal inference by measuring and modeling key time-varying covariates (e.g., major life events, school climate/stress indicators, and family changes), using more frequent assessments, or combining RI-CLPM with designs that better account for time-varying confounding (e.g., planned interventions or quasi-experimental approaches).

## 5. Conclusions

This study provides longitudinal evidence for the distinct roles of psychological capital and perceived social support in shaping problem behavior among rural Chinese adolescents. By applying random-intercept cross-lagged panel models, the present findings demonstrate that associations observed in traditional cross-lagged models largely reflect stable between-person differences, whereas within-person processes reveal more nuanced and developmentally specific patterns.

Psychological capital showed a time-specific within-person protective effect, with short-term increases predicting subsequent reductions in problem behavior during early adolescence. In contrast, perceived social support exhibited more robust and dynamic within-person associations, characterized by both protective effects on problem behavior and reciprocal erosion following behavioral difficulties. Together, these findings suggest that internal psychological resources and external social resources contribute to adolescent behavioral adjustment through complementary mechanisms.

From a practical perspective, interventions for rural adolescents should adopt a dual-focus strategy: fostering psychological capital early in adolescence while simultaneously strengthening and safeguarding social support networks. Such an approach acknowledges both the internal capacities adolescents bring to developmental challenges and the relational environments that sustain adaptive functioning over time. In practice, this dual-focus strategy could be operationalized through low-cost, school-based delivery (e.g., brief PsyCap modules during class meetings) alongside routine teacher mentoring and peer-support activities, supplemented by simple home–school communication protocols where caregivers or migrant parents can be reached by phone/online.

## Figures and Tables

**Table 1:** Distribution of Participants (*n* = 770).

Variable	Category	*n*	%
Gender	Male	374	49.86%
	Female	396	50.14%
Grade	7th grade	204	25.86%
	8th grade	207	24.36%
	10th grade	183	24.00%
	11th grade	176	25.78%

**Table 2:** Correlations Among Problem Behaviors, Psychological Capital, and Perceived Social Support Across Different Stages.

	1.1	1.2	1.3	2.1	2.2	2.3	3.1	3.2	3.3
1.1	1								
1.2	−0.76 **	1							
1.3	−0.64 **	0.76 **	1						
2.1	0.40 **	−0.32 **	−0.26 **	1					
2.2	−0.44 **	0.55 **	0.43 **	−0.55 **	1				
2.3	−0.41 **	0.47 **	0.51 **	−0.49 **	0.83 **	1			
3.1	0.32 **	−0.26 **	−0.21 **	0.49 **	−0.44 **	−0.42 **	1		
3.2	−0.40 **	0.47 **	0.39 **	−0.41 **	0.66 **	0.63 **	−0.71 **	1	
3.3	−0.33 **	0.38 **	0.41 **	−0.34 **	0.58 **	0.66 **	−0.65 **	0.85 **	1
*M*	7.98	130.68	65.00	4.95	134.39	65.72	5.91	125.48	62.51
*SD*	5.23	24.13	12.62	2.84	25.75	13.75	4.38	31.49	16.16

Note: 1.1 Problem Behavior (Time 1); 1.2 Psychological Capital (Time 1); 1.3 Perceived Social Support (Time 1); 2.1 Problem Behavior (Time 2); 2.2 Psychological Capital (Time 2); 2.3 Perceived Social Support (Time 2); 3.1 Problem Behavior (Time 3); 3.2 Psychological Capital (Time 3); 3.3 Perceived Social Support (Time 3). *M* = mean; *SD* = standard deviation. ** *p* < 0.01.

## Data Availability

The data presented in this study are available on request from the corresponding author. The data are not publicly available due to privacy and ethical restrictions.
